# Errors in Diagnosis

**Published:** 1851-03

**Authors:** C. H. Cleaveland

**Affiliations:** Waterbury, Vt.


*Errors in Diagnosis.* By C. H. Cleaveland, M. D., of Waterbury, Vt.—Perhaps in no other way could the younger members of our profession be more benefited, than to have pointed out to them the errors that have occurred by a too hasty and incomplete investigation of cases before deciding upon the nature of the case, and the course of treatment necessary to pursue to ensure a cure.

Some years since a child who was playing with a *button* put it into his mouth, *and he said he had swallowed it;* but that night he was taken with a hoarseness somewhat resembling croup, and the parents becoming frightened, sent for their physician, who, after listening to the child's breathing, discovered that the button was lodged at the bifurcation of the trachea, or at the commencement of the bronchial tubes.

He called an elderly physician, his former preceptor, in consultation, who confirmed the diagnosis, and at once they resorted to tracheotomy, but did not that day succeed in dislodging the button, although they thought they could dintinctly feel it. The next day they reopened the orifice, but with no better success.

After a few days, the button was found in the boy's fæces, but he entirely lost his voice, and the last I heard of him he remained mute.

Several years since, a little school-girl fell from a tree, where she had climbed to procure some twigs, and her injury was so great that a physician was sent for, who discovered that she had broken her thigh, at the upper third; and after she was carried to her home, he reduced the fracture, and made the proper dressings, enjoining that the limb should not be moved, or the splints displaced until his next visit. The second day, when he called, the little patient was not in bed; and, on looking out of the window, he discovered her feeding the chickens in the farm-yard.

The first year of my practice, a girl, aged about fourteen, was attacked with the epidemic erysipelas at the time of her first menstruation. The disease located itself upon the uterus, and soon her abdomen was as large as a mother's at six months' advance in pregnancy. One day, an old physician, hearing I had a difficult case, volunteered a call, and after a proper examination, at first pronounced her difficulty a suppression of urine; but in that he was overruled by the mother, who gave him ocular proof of his error. Next he said there was an abscess forming in the flesh of the lower part of the abdomen, and had a large poultice applied, *to draw it to a head.*

In a neighboring town, a man had been sick several days with a peculiar pain, and strange swelling of the abdomen; and the two medical attendants were doing all they could with fomentations, poultices, etc., to induce suppuration, when my friend Dr. B. was called in. The *catheter* gave him immediate relief.

I could fill pages with similar errors, but think these will answer as well as a larger number.—*N. H. Journal of Medicine.*

